# Prognostic Implication of pAMPK Immunohistochemical Staining by Subcellular Location and Its Association with SMAD Protein Expression in Clear Cell Renal Cell Carcinoma

**DOI:** 10.3390/cancers11101602

**Published:** 2019-10-21

**Authors:** Minsun Jung, Jeong Hoon Lee, Cheol Lee, Jeong Hwan Park, Yu Rang Park, Kyung Chul Moon

**Affiliations:** 1 Department of Pathology, Seoul National University Hospital, Seoul 03080, Korea; jjunglammy@gmail.com (M.J.); fejhh@hanmail.net (C.L.); 2 Department of Biomedical Systems Informatics, Yonsei University College of Medicine, Seoul 03722, Korea; sosal@snu.ac.kr (J.H.L.); YURANGPARK@yuhs.ac (Y.R.P.); 3 Department of Pathology, SMG-SNU Boramae Medical Center, Seoul 07061, Korea; hopemd@hanmail.net; 4 Kidney Research Institute, Medical Research Center, Seoul National University College of Medicine, Seoul 03080, Korea

**Keywords:** clear cell renal cell carcinoma, AMP-activated protein kinases, immunohistochemistry, prognosis, SMAD proteins, transforming growth factor beta

## Abstract

Although cytoplasmic AMP-activated protein kinase (AMPK) has been known as a tumor-suppressor protein, nuclear AMPK is suggested to support clear cell renal cell carcinoma (ccRCC). In addition, pAMPK interacts with TGF-β/SMAD, which is one of the frequently altered pathways in ccRCC. In this study, we investigated the prognostic significance of pAMPK with respect to subcellular location and investigated its interaction with TGF-β/SMAD in ccRCC. Immunohistochemical staining for pAMPK, pSMAD2 and SMAD4 was conducted on tissue microarray of 987 ccRCC specimens. Moreover, the levels of pSMAD2 were measured in Caki-1 cells treated with 5-aminoimidazole-4-carboxamide ribonucleotide. The relationship between *AMPK*/pAMPK and *TGFB1* expression was determined using the TCGA database. As a result, pAMPK positivity, either in the cytoplasm or nuclei, was independently associated with improved ccRCC prognosis, after adjusting for TNM stage and WHO grade. Furthermore, pAMPK-positive ccRCC displayed increased pSMAD2 and SMAD4 expression, while activation of pAMPK increased pSMAD2 in Caki-1 cells. However, *AMPK*/pAMPK expression was inversely correlated with *TGFB1* expression in the TCGA database. Therefore, pAMPK immunostaining, both in the cytoplasm and nuclei, is a useful prognostic biomarker for ccRCC. pAMPK targets TGF-β-independent phosphorylation of SMAD2 and activates pSMAD2/SMAD4, representing a novel anti-tumoral mechanism of pAMPK in ccRCC.

## 1. Introduction

Clear cell renal cell carcinoma (ccRCC), the most common type of renal cell carcinoma, is characterized by genetic alterations that regulate cellular metabolism [1,2]. For example, accumulation of an oxygen-sensing protein, hypoxia-inducible factor (HIF)-α, by the mutational loss of the *VHL* tumor-suppressor gene supports ccRCC progression via angiogenesis and epithelial-mesenchymal transition (EMT) [2]. Another metabolic hallmark of ccRCC is a change in the glucose-sensing machinery caused by constitutive activation of the *PI3K*/*AKT*/*MTOR* pathway [1,2]. Activated mammalian target of rapamycin complex 1 (mTORC1) also stimulates development and progression of ccRCC, indicating that mTORC1 is a target for the treatment of metastatic ccRCC [1,2,3]. Furthermore, lipogenic metabolism is one of the important biologic signatures of ccRCC [2,4,5]. Consistent with these findings, altered expression of metabolism-associated molecules in ccRCC, including AMP-activated protein kinase (AMPK), has been reported to be significantly associated with clinical outcomes [1,6].

AMPK is an intracellular metabolic switch that increases catabolic processes upon activation by threonine (T172) phosphorylation; this phosphorylation is mediated by liver kinase B1 (LKB1) and is stimulated by an increased AMP:ATP ratio [7,8]. pAMPK has been highlighted for its roles as both a metabolic regulator and a tumor suppressor [7]. For example, the AMPK activator 5-aminoimidazole-4-carboxamide ribonucleotide (AICAR) has been shown to inhibit mTORC1 and thus negatively regulate proliferation and survival of multiple types of cancer cells, including ccRCC [7,9]. In agreement with these findings, increased AMPK/pAMPK expression is indicative of favorable survival in patients with carcinomas of the uterine cervix [10], ovary [11], and liver [12] whose tumor cells display cytoplasmic AMPK/pAMPK immunohistochemical (IHC) staining. Conversely, and paradoxically, nuclear pAMPK has been revealed to promote the survival, proliferation, and metastatic capacity of malignant cells under metabolic stress, likely through oncogene activation [13,14]. Recently, Liu et al. [14] demonstrated that nuclear pAMPK mediates the proliferation of glucose-deprived human renal cell carcinoma cells, by recruiting pyruvate kinase isozymes M2 and β-catenin. Although analysis from ccRCC tumor lysates revealed that increased AMPK mRNA and pAMPK are associated with favorable outcomes [1,6], the prognostic significance of pAMPK subcellular location has not yet been investigated in patients with ccRCC.

pAMPK has also been shown to attenuate signaling transduction via the transforming growth factor-β (TGF-β)/SMAD pathway in numerous non-neoplastic cells by inhibiting phosphorylation of SMAD2/SMAD3 or nuclear translocation of SMAD4 [15,16,17,18,19]. In cancer, the connection between pAMPK and TGF-β/SMAD has been poorly investigated, with the exception of one study in breast cancer cells which revealed that pAMPK decreased invasion via downregulation of TGF-β/SMAD-dependent EMT [20]. Upon receptor-regulated phosphorylation by TGF-β, pSMAD2/pSMAD3 forms a complex with SMAD4 and acts as a coactivator of numerous TGF-β target promoters in the nuclei that contributes to either tumorigenesis or tumor progression, depending on the context [21]. In light of the fact that nuclear SMAD2/SMAD3/SMAD4 expression was significantly associated with ccRCC prognosis [22], SMAD proteins may be targets of pAMPK in regulating the behavior of ccRCC. In this study, we aimed to clarify the prognostic significance of pAMPK with respect to the subcellular location in ccRCC, as revealed by IHC staining. In addition, we tried to determine whether the expression of SMAD proteins was governed by pAMPK in ccRCC.

## 2. Results

### 2.1. Patients and pAMPK IHC Staining

The demographic and clinicopathological characteristics of the discovery and validation of ccRCC patient cohorts are summarized in Table 1. In the discovery and validation cohorts, the male-to-female sex ratios were 2.8 and 3.0 and the median ages were 58 (range, 20–81) and 56 (range, 24–84) years, respectively.

**Figure 1:**
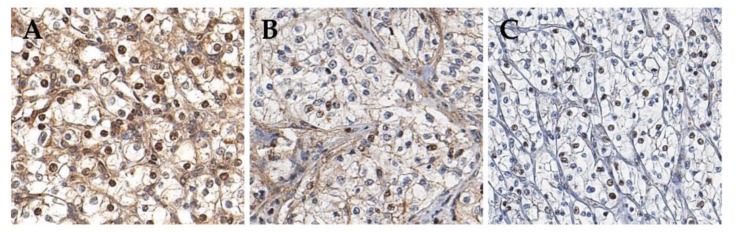
Immunohistochemical staining for pAMPK. (**A**) More than 50% of tumor cells expressed pAMPK both in the cytoplasm and nuclei; (**B**) More than 50% of tumor cells are stained for pAMPK in the cytoplasm but less than 10% of tumor cells show nuclear staining for pAMPK; (**C**) More than 50% of the tumor cells exhibits pAMPK only in the nuclei.

IHC staining revealed cytoplasmic (Figure 1A,B) and nuclear (Figure 1B,C) pAMPK positivity in 250 (55.2%) and 228 (50.3%) samples, respectively, of the discovery cohort, and in 242 (45.3%) and 231 (43.3%) patients, respectively, of the validation cohort (Table 1). The results of this dichotomous assessment of cytoplasmic and nuclear pAMPK expression, described in detail in *Materials and Methods 4.3.*, showed high agreement between the tissue microarray (TMA) and matched whole-section slides from 10 randomly selected patients. Both cytoplasmic and nuclear pAMPK positivity was significantly associated with small tumor size (*p* < 0.001), low TNM stage (*p* < 0.001), and low WHO grade (*p* < 0.001) in the discovery cohort, which was verified in the validation cohort (Table 1).

### 2.2. Positive IHC Staining for pAMPK Was Significantly Associated with Improved ccRCC Prognosis

**Figure 2:**
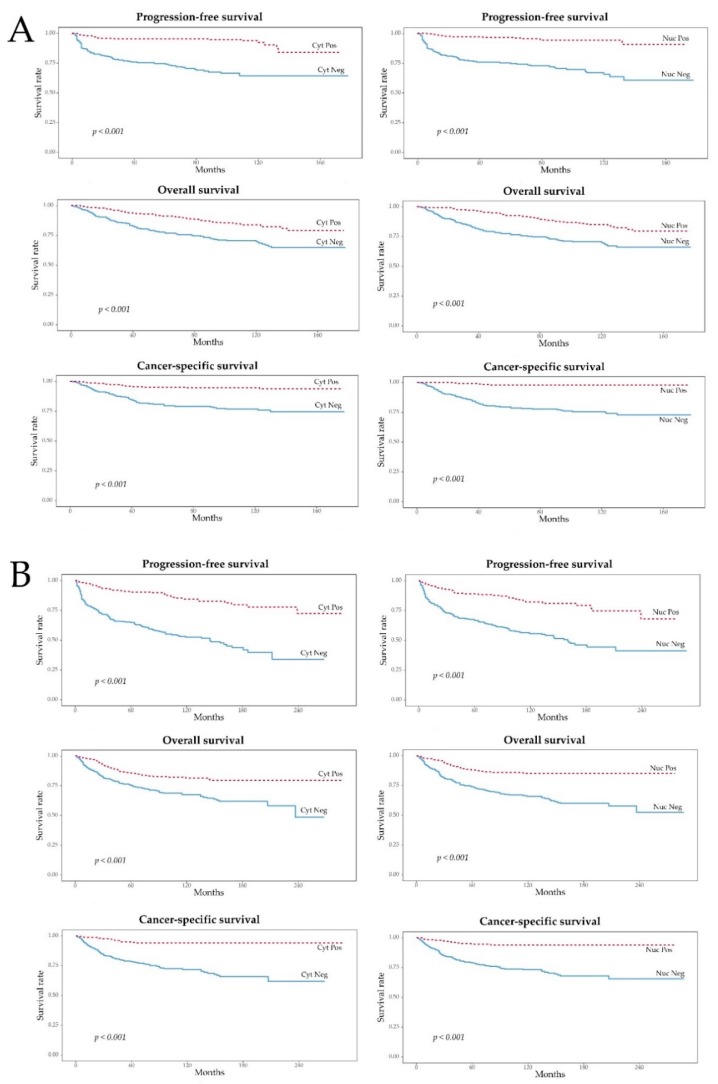
Survival analyses of pAMPK cytoplasmic and nuclear expression. (**A**) Discovery cohort; (**B**) Validation cohort. Cyt, cytoplasm; Pos, positive; Neg, negative; Nuc, nucleus.

The median follow-up periods from the discovery and validation cohorts were 121 (range, 1–178) and 102 (range, 2–288) months, respectively. The median overall survival (OS) was not reached in either cohort. Kaplan–Meier and log-rank tests showed that pAMPK-positive ccRCC, either in the cytoplasm or in the nuclei, was associated with longer progression-free survival (PFS) (*p* < 0.001), overall survival (OS) (*p* < 0.001), and cancer-specific survival (CSS) (*p* < 0.001) than pAMPK-negative ccRCC, both in the discovery and validation cohorts (Figure 2). Univariate Cox regression analysis showed that patients with pAMPK-positive ccRCC (with either a cytoplasmic or nuclear pattern) were less likely to experience disease progression (*p* < 0.001), death (*p* < 0.001), and disease-specific death (*p* < 0.001); these findings were true in both the discovery (Table 2) and validation (Table 3) cohorts. A multivariate analysis incorporating TNM stage and WHO grade showed that pAMPK positivity is an independent prognostic factor for favorable PFS (cytoplasmic, hazard ratio [HR] = 0.260, *p* < 0.001; nuclear, HR = 0.308, *p* < 0.001), OS (cytoplasmic, HR = 0.656, *p* = 0.032), and CSS (cytoplasmic, HR = 0.374, *p* = 0.001; nuclear, HR = 0.232, *p* = 0.003) in the discovery cohort (Table 2). The prognostic significance of cytoplasmic and nuclear pAMPK expression adjusted for TNM stage and WHO grade was confirmed in the validation cohort (Table 3).

### 2.3. pAMPK Induced Nuclear SMAD Protein Expression in ccRCC

**Figure 3:**
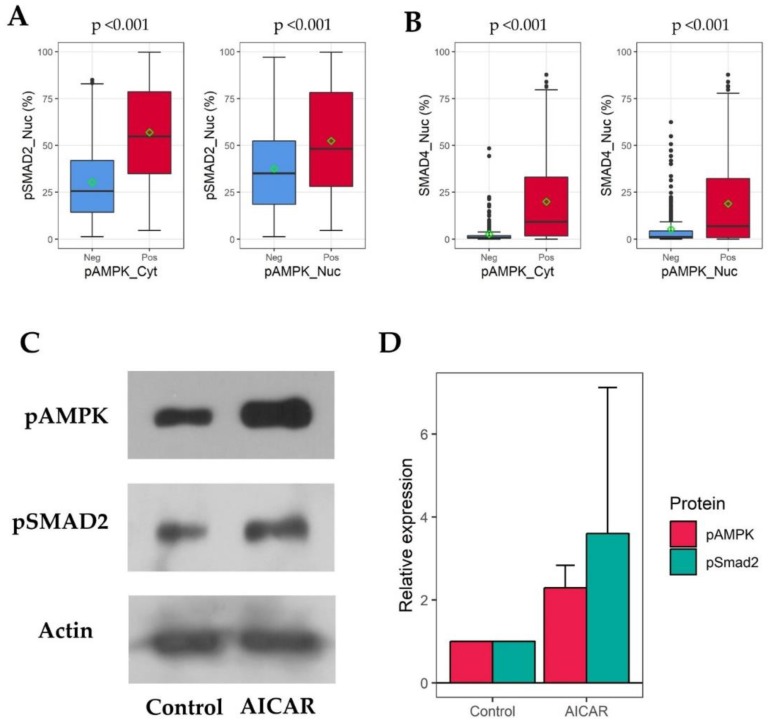
pSMAD2/SMAD4 upregulation by pAMPK in ccRCC. pAMPK-positive ccRCC, either in the cytoplasm or nuclei, shows higher nuclear expression levels of (**A**) pSMAD2 and (**B**) SMAD4 than pAMPK-negative ccRCC (Mann–Whitney U tests). Green square indicates the mean value; (**C**) AICAR activates pAMPK and induces pSMAD2 expression in Caki-1 cells; (**D**) The relative intensity of Western blot results before (control) and after AICAR treatment is shown as a mean (bar) with a standard deviation (line) (Rex 3.0.4, RexSoft Inc., Seoul, Korea).

We compared nuclear pSMAD2 and SMAD4 immunoreactivity of pAMPK-positive and pAMPK-negative tumors. The mean (± standard deviation) pSMAD2 expression was 57.0% (±26.2) and 30.4% (±19.7) (*p* < 0.001) in the cytoplasmic pAMPK-positive and pAMPK-negative ccRCC samples, respectively, and 52.5% (± 28.5) and 37.5% (± 23.0) (*p* < 0.001) in the nuclear pAMPK-positive and pAMPK-negative ccRCC specimens, respectively. Similarly, SMAD4 nuclear expression was observed in 20.0% (±22.6) and 2.6% (±5.9) (*p* < 0.001) of tumor cells with cytoplasmic pAMPK positivity and negativity, respectively, and in 19.0% (±23.2) and 5.4% (±10.5) (*p* < 0.001) of those with positive and negative nuclear pAMPK immunostaining, respectively (Figure 3B). We next sought to determine whether pAMPK positively regulates SMAD protein expression. AICAR treatment activated pAMPK and increased pSMAD2 expression in Caki-1 cells (Figure 3C,D).

**Figure 4:**
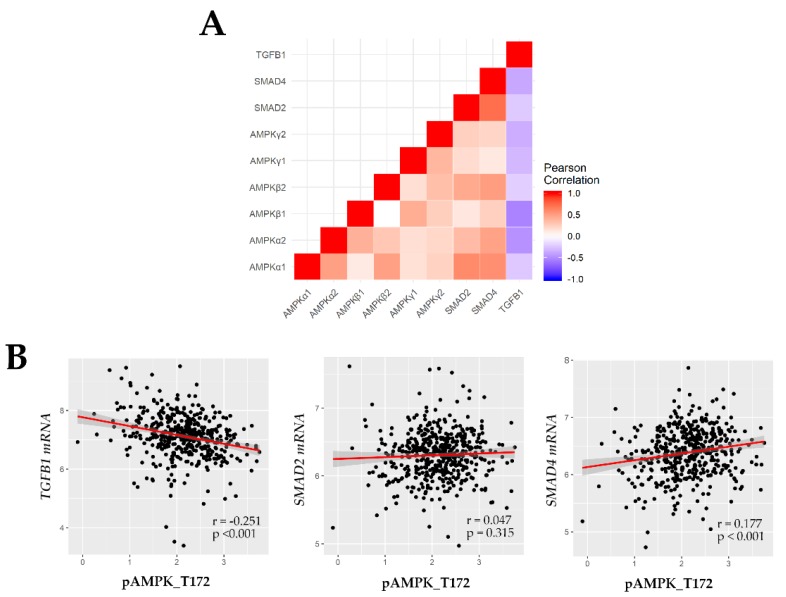
Correlation coefficient among *TGFB1*, *SMAD2*, *SMAD4*, and AMPK (mRNA and T172 phosphoprotein) analyzed from the TCGA ccRCC database. (**A**) *TGFB1* is inversely but *SMAD2* and *SMAD4* are positively correlated with AMPK mRNA in the TCGA mRNA database; (**B**) Similar trends are identified between *TGFB1*/*SMAD2*/*SMAD4* (TCGA mRNA database) and pAMPK^T172^ protein (TCGA reverse phase protein array data of the matched samples).

Next, we asked whether the pAMPK-mediated SMAD induction was dependent on the TGF-β/SMAD pathway. To this end, we investigated the correlation between AMPK mRNA (*AMPKα1*, *AMPKα2*, *AMPKβ1*, *AMPKβ2*, *AMPKγ1*, and *AMPKγ2*) and phosphoprotein (T172) levels, and the mRNA expression levels of TGF-β (*TGFB1*) and SMAD (*SMAD2*, *SMAD4*) in the TCGA ccRCC database. Our analysis revealed that levels of AMPK mRNA and pAMPK were inversely correlated with *TGFB1* but were weakly and positively correlated with expression of *SMAD2* and *SMAD4* (Figure 4). Therefore, both cytoplasmic and nuclear pAMPK-positive ccRCC was enriched for nuclear pSMAD2 and SMAD4. Further, pAMPK induced phosphorylation of SMAD2 in a TGF-β-independent manner.

## 3. Discussion

Here, we have revealed the prognostic implication of IHC staining for pAMPK in large cohorts of ccRCC patients. pAMPK positivity was related to low-risk pathological traits of ccRCC, including small tumor size, early TNM stage, and low WHO grade, and was an independent favorable prognostic factor in ccRCC. These results were consistent across samples with cytoplasmic and/or nuclear pAMPK IHC staining, suggesting that IHC staining for pAMPK may be a useful prognostic biomarker for ccRCC. Furthermore, pAMPK-positive ccRCC showed higher levels of nuclear pSMAD2 and SMAD4 expression than those in pAMPK-negative tumors. Consistent with this result, Caki-1 cells treated with AICAR showed increased expression of pSMAD2. *SMAD2*/*SMAD4* mRNA also showed a modestly positive correlation with AMPK mRNA/phosphoprotein, whereas *TGFB1* showed an inverse correlation, suggesting that pAMPK activates SMAD expression through a non-TGF-β pathway.

pAMPK inhibits growth, invasion, EMT, and metastasis by modulating various signaling axes, including downregulation of mTORC1, HIF, and NF-κB and upregulation of Forkhead box class O (FoxO)3a and p53 [7,12,23,24,25]. In addition, pAMPK is responsible for restricting ATP-consumption by directly suppressing fatty acid synthesis enzymes, such as acetyl-CoA carboxylase and fatty acid synthase. In other words, pAMPK shifts metabolism away from the Warburg effect-like status, an environment that is supportive of ccRCC progression [1,6,7,24]. Our clinical results as well as previous preclinical data propose that AMPK activation may be useful as a ccRCC treatment strategy [25,26]. pAMPK expression may affect the treatment response to targeted agents in ccRCC. For example, rapamycin, an inhibitor to mammalian target of rapamycin, displayed additive counteraction against the invasion and growth of renal cell carcinoma when this was administered together with AICAR [25]. In addition, because pAMPK inhibits HIF-α activity and HIF-α stably upregulates vascular endothelial growth factor in ccRCC [2,25], the association between pAMPK expression and vascular endothelial growth factor receptor tyrosine kinase inhibitors might be worth investigating. However, the subcellular location-specific tumor-suppressive functions of AMPK have been controversial. For example, nuclear pAMPK increased transcription of pro-cancerous molecules, such as cyclin D1, c-myc, and Oct4, in cancer cells under metabolic stresses (e.g., in glucose-deprived conditions) [13,14]. The clinical significance of this nuclear pAMPK immunoreactivity has never been investigated in vivo because prior studies have reported that IHC staining for pAMPK is confined to the cytoplasm of normal and neoplastic human tissue [10,11,12,27,28]. In the ccRCC TCGA reports, the prognostic significance of pAMPK was demonstrated using protein array, without considering pAMPK subcellular localization [1,6]. We observed that both cytoplasmic and nuclear pAMPK is independently associated with favorable prognoses in ccRCC. Previous studies have revealed that nuclear pAMPK can function in either an inhibitory or a stimulatory manner in renal cell carcinoma, depending on context: the oncogenic function of nuclear AMPK is confined to glucose-deprived renal cell carcinoma cells, whereas regular tumor-suppressive functions predominate under usual conditions [14]. Therefore, it is reasonable to speculate that the hostile environments that encourage nuclear pAMPK to stimulate ccRCC progression were present in only a restricted number of patients in this study. In addition, it is noteworthy that glucose starvation can also lead to the activation of various signals other than AMPK, including Akt and ERK [23].

The TGF-β/SMAD signaling pathway, a major regulator of carcinogenesis and the progression of various tumor types [29], is frequently altered in ccRCC [1]. In fact, pAMPK has been shown to inhibit SMAD-mediated TGF-β signal transduction through various mechanisms, by which pAMPK exhibits anti-fibrogenic and anti-EMT functions [15,16,17,18,20]. For example, pAMPK prevented TGF-β-mediated phosphoactivation of SMAD2 and/or SMAD3 in vascular smooth muscle and breast cancer cells [17,20]. In mesenchymal cells, pAMPK also reduced the stability and nuclear localization of SMAD4 [18] and the interaction of SMAD3 with its coactivator, p300, without affecting the phosphorylation of SMAD2 or SMAD3 [16]. In contrast, we demonstrated that pAMPK-positive ccRCC showed higher levels of nuclear pSMAD2 and SMAD4 than those in pAMPK-negative tumors. We hypothesize that this positive relationship between pAMPK and pSMAD2/SMAD4 may be involved in an important, as-yet-unidentified mechanism that is critical to the inhibition of ccRCC. In addition, this may explain a previous report that ccRCC displaying nuclear SMAD2/SMAD3/SMAD4 protein expression had, for unknown reasons, favorable outcomes [22]. It is known that LKB1, kinase upstream of pAMPK, dictates phosphoactivation of AMPK, except on limited occasions when AMPK might be activated by calcium/calmodulin-dependent protein kinase kinase 2, TGF-β-activated kinase 1, or AMP through phosphorylation or allosteric modification [7,8]. AICAR is an AMP-mimetic that directly binds to AMPK and facilitates its phosphorylation by LKB1 [7,19]. Therefore, it is safe to say that LKB1 played a critical role in activating pAMPK in this study. LKB1 may directly increase TGF-β signaling in an AMPK-independent way, as suggested in other studies [30,31]. Nevertheless, we identified a negative correlation between *TGFB1* and both AMPK mRNA and pAMPK proteins in the TCGA ccRCC database, in agreement with a previous report [20]. Low *TGFB1* mRNA levels are likely attributable to an attenuated TGF-β/SMAD cascade [31]. The positive correlation between *SMAD2*/*SMAD4* mRNA levels and *AMPK*/pAMPK in the TCGA database was weak and inconsistent, compared to the tight correlation between pSMAD2/SMAD4 and pAMPK expression in our TMA and Western blot analyses. Therefore, we hypothesize that pAMPK post-transcriptionally or post-translationally activates pSMAD2/SMAD4 by phosphorylating SMAD2 via a TGF-β-independent mechanism. Furthermore, the results suggest that pAMPK probably conducts a tumor-suppressive function through pSMAD2/SMAD4 activation, at least in part. Upregulation of nuclear FoxO3a may connect the coactivation of pAMPK and SMAD. FoxO3 is also a good candidate for an interdependent molecule mediating the antitumoral functions of pAMPK and pSMAD2/SMAD4 in ccRCC. In ovarian cancer, FoxO3a interacted with a SMAD2/SMAD3/SMAD4 complex in the nucleus, which could maintain activated SMAD proteins at high levels, and this interaction in turn promoted the cell-cycle arrest synergistically [32,33]. In addition, previous studies showed that the activation of AMPK increased both the nuclear localization and stability of FoxO3a in cancer and enhanced the transcription of autophagy-associated genes, leading to cell death [23,34]. Taken together, this putative pAMPK/FoxO3/SMAD interaction may lead to favorable outcomes in pAMPK-positive ccRCC with high expression of SMAD proteins. Presumably, this is a novel biological function of pAMPK/pSMAD2/SMAD4 in ccRCC that warrants further studies. Secondly, given that AMPK is a kinase of diverse targets and the TGF-β/SMAD pathway engages in a reciprocal cross talk with various molecules [7,21], it is readily assumable that pAMPK might activate and cooperate with pSMAD2/SMAD4 to downregulate ccRCC, either directly or indirectly by way of modulating other pathways.

There are a few limitations in this study. Although it is well known that pSMAD2 and pSMAD3 are very similar proteins that can form a complex with SMAD4 to activate transcriptional responses [29], the interaction between pSMAD3 and pAMPK was not investigated in this study. Along with the positive correlation between pAMPK and nuclear pSMAD2/SMAD4 in IHC, the Western blot result puts forward the idea that pAMPK overexpression phosphorylates SMAD2 and subsequently activates pSMAD2/SMAD4 in ccRCC. However, more detailed in vitro studies adopting a constitutively active mutant of *AMPK* or depletion of pAMPK using compound C or siRNA to *AMPK* would profoundly improve the understanding of the causal relationship between pAMPK and pSMAD2/SMAD4 activation. In addition, despite the unequivocal overexpression of pSMAD2 and SMAD4 in pAMPK-positive tumors, it still remains uncertain whether pAMPK directly induces SMAD-mediated transcriptional activities in ccRCC. Previous reports demonstrated that SMAD activities were regulated by pAMPK at the transcriptional level in various conditions [15,16,20], which accompanied increased expression of the pAMPK and AMPKα2 subunit in the nuclei of human mesangial cells as well [15]. Although these results imply that pAMPK may directly associate with SMAD proteins and modulate their transcriptional function in the nuclei [15], this phenomenon has not been verified in ccRCC. Further investigation will be required to elucidate the detailed functional effects of pAMPK-induced activation of SMAD proteins, including SMAD-responsive transcription and the regulation of its downstream targets.

## 4. Materials and Methods

### 4.1. Patients’ Cohorts

In total, 987 ccRCC samples were surgically resected at the Seoul National University Hospital between 1995 and 2008, and separated into discovery (*n* = 453; 2003–2008) and validation (*n* = 534; 1995-2004) cohorts. Of these samples, 294 cases of the discovery cohort and 493 cases of the validation cohort were radical nephrectomy specimens, while the others were partial nephrectomy specimens. Each tumor sample was reviewed for histologic type, TNM stage, and WHO grade. The TNM stage was reclassified according to the American Joint Committee on Cancer—Cancer Staging Manual, 8th edition [35]. Clinical data were obtained from medical records. Patients who received neoadjuvant treatment, displayed bilateral disease at the time of diagnosis, or who had Von Hippel–Lindau syndrome were excluded. This study was approved by the Institutional Review Board of Seoul National University Hospital (H-1810-150-983).

### 4.2. TMA Construction and IHC Staining

Two cores (2 mm in diameter) from each specimen were embedded in recipient paraffin blocks using a trephine apparatus (Superbiochips Laboratories, Seoul, Republic of Korea) for TMA construction. IHC staining was conducted on 4-μm-thick TMA sections using the Benchmark autostainer (Ventana, Tucson, AZ) according to the manufacturer’s instructions. IHC staining was conducted using a rabbit monoclonal antibody against pAMPK^T172^ (1:100; Cat. #2535; Cell Signaling Technology, Danvers, MA), a rabbit polyclonal antibody against pSMAD2^S467^ (1:70; Cat. #ab53100; Abcam, Cambridge, UK), and a mouse monoclonal antibody against SMAD4 (1:100; Cat. #sc-7966; Santa Cruz Biotechnology). One TMA slide was stained without primary anti-pAMPK antibody as a negative control.

### 4.3. Establishment of Cut-Off Criteria for pAMPK IHC Staining Positivity

pAMPK expression was observed in the cytoplasm and the nuclei of tumor cells (Figure 1). Firstly, the extent of tumor cells in the discovery cohort displaying at least moderate immunoreactivity was assessed semiquantitatively as follows: <10%, 10–50%, and >50%. Based on both sample distribution and prognostic significance, 10% staining was defined as the best cut-off value for both cytoplasmic and nuclear pAMPK positivity (Figure S1). Next, the prognostic significance of pAMPK staining was confirmed in the validation cohort using the same cut-off criteria. Regarding discordant cases from duplicate TMA cores, the lower pAMPK expression level was used. To account for intratumoral heterogeneity, IHC staining for pAMPK was also conducted on whole sections from 10 randomly selected cases in the discovery cohort. The percentage of tumor cells (%) positive for nuclear pSMAD2 and SMAD4 was calculated from both TMA cores, using QuPath version 0.1.2 [36] on digitally scanned slides (Aperio AT2, Leica Biosystem, Wetzlar, Germany). The mean values of pSMAD2 and SMAD4 are shown.

### 4.4. Western Blot Analysis

A human ccRCC cell line, Caki-1, was obtained from the Korean Cell Line Bank (KCLB, Seoul, Republic of Korea) and was cultured in Dulbecco’s Modified Eagle’s Medium (DMEM) supplemented with 10% fetal bovine serum in a 5% CO_2_ humidified incubator. To evaluate the AMPK/SMAD2 signaling pathway, we used the AMPK activator, AICAR (Sigma, St. Louis, MO, USA). Caki-1 cells were treated with 1 mM AICAR for 6 hours, and then, the cells were harvested for Western blot analysis. Cell lysates were electrophoretically resolved on a 10% polyacrylamide gel in a sodium dodecyl sulfate buffer and then transferred onto nitrocellulose membranes. Afterward, the blots were incubated with antibodies against pAMPK^T172^ (Cat. #2535, Cell Signaling Technology) and pSMAD2^S465/467^ (Cat. #3108; Cell Signaling Technology). These experiments were conducted in triplicate and are presented as the mean ± standard deviation.

### 4.5. Characteristics of the TCGA ccRCC Dataset

ccRCC mRNA sequencing and reverse phase protein array data with clinicopathological information generated by the TCGA Research Network were obtained using the Broad Institute GDAC Firehose [37]. The mRNA sequencing dataset was generated from 552 primary ccRCC samples and quantified by RSEM (RNA-Seq by Expectation) [38]. The median age in this sample was 61 years (range, 26–90) and the male-to-female sex ratio was 1.8:1. Approximately, 60% (316/522) and 46% (236/514) of these patients had a low TNM stage (I or II) and low Furhman grade (1 or 2), respectively.

We next constructed a design matrix using the DGEList function in the EdgeR module. To filter out genes with low expression levels, we excluded those genes with counts per million (cpm) values <1 in at least half of the samples [39]. As a result of this filtering, *AMPKγ3* (*PRKAG3*) was omitted. RSEM read counts underwent Trimmed Mean of M-values (TMM) normalization and logCPM transformation using voom [40]. Of the patients whose samples were included in the mRNA dataset, 472 also had reverse phase protein array information, including pAMPK^T172^ protein expression levels. These data were analyzed to examine the correlation between *AMPK*/pAMPK^T172^ and *TGFB1*/*SMAD* mRNA expression. 

### 4.6. Statistical Analysis

The interrelation between pAMPK IHC staining and clinicopathological characteristics was analyzed using Pearson’s χ^2^ test with Yates’ correction for categorical variables and with the Mann–Whitney test for continuous variables. The PFS period was calculated as the interval between surgery and recurrence, progression, metastasis, or the last follow-up visit. The OS duration was defined as the period between surgery and death from any cause or the last follow-up. The CSS duration was defined as the interval between surgery and cancer-related death or the last follow-up visit. Kaplan–Meier analysis and the log-rank test were used to compare survival. A Cox proportional hazard regression model was used for univariate and multivariate survival analyses. The strength and direction of the linear relationships among *AMPK*/pAMPK, *TGFβ1*, and *SMAD2*/*SMAD4* expression was assessed using Pearson’s r test. All statistical analyses were performed using SPSS Statistics 25 (IBM Co., Armonk, NY) or in R, with a 2-tailed *p* < 0.05 considered statistically significant.

## 5. Conclusions

Both cytoplasmic and nuclear pAMPK immunostaining are independently associated with favorable outcomes in ccRCC. pAMPK positivity is associated with nuclear overexpression of pSMAD2 and SMAD4, through stimulation of TGF-β-independent phosphorylation of SMAD2, which is a novel antitumoral mechanism for pAMPK in ccRCC. Understanding the interaction between pAMPK and SMAD proteins will facilitate the use of AMPK activation as a strategy for ccRCC treatment.

## Figures and Tables

**Table 1:** pAMPK expression and clinicopathological details of the discovery and validation cohorts.

Discovery Cohort	Cyt Pos ^1^	Cyt Neg	*p*	Nuc Pos ^1^	Nuc Neg	*p*	Total
Number	*n* = 250 (55.2%)	*n* = 203 (44.8%)		*n* = 228 (50.3%)	*n* = 225 (49.7%)		453
Age (year)			0.006			0.009	
≥58	118 (47.2%)	123 (60.6%)		107 (46.9%)	134 (59.6%)		241 (53.2%)
<58	132 (52.8%)	80 (39.4%)		121 (53.1%)	91 (40.4%)		212 (46.8%)
Sex			0.483			0.003	
Male	180 (72.0%)	153 (75.4%)		153 (67.1%)	180 (80.0%)		333 (73.5%)
Female	70 (28.0%)	50 (24.6%)		75 (32.9%)	45 (20.0%)		120 (26.5%)
Size (cm)^2^	3.0 [2.0–4.5]	5.0 [3.0–7.9]	<0.001 ^3^	3.0 [2.0–4.7]	4.5 [3.0–7.5]	<0.001 ^3^	3.5 [2.3–6.0]
TNM stage			<0.001			<0.001	
Low (I or II)	219 (87.6%)	138 (68.0%)		209 (91.7%)	148 (65.8%)		357 (78.8%)
High (III or IV)	31 (12.4%)	65 (32.0%)		19 (8.3%)	77 (34.2%)		96 (21.2%)
WHO grade			<0.001			<0.001	
Low (1 or 2)	141 (56.4%)	79 (38.9%)		155 (68.0%)	65 (28.9%)		220 (48.6%)
High (3 or 4)	109 (43.6%)	124 (61.1%)		73 (32.0%)	160 (71.1%)		233 (51.4%)
**Validation cohort**	**Cyt Pos ^1^**	**Cyt Neg**	***p***	**Nuc Pos ^1^**	**Nuc Neg**	***p***	**Total**
Number	*n* = 242 (45.3%)	*n* = 292 (54.7%)		*n* = 231 (43.3%)	*n* = 303 (56.7%)		534
Age (year)			0.078			0.045	
≥56	114 (47.1%)	161 (55.1%)		107 (46.3%)	168 (55.4%)		275 (51.5%)
<56	128 (52.9%)	131 (44.9%)		124 (53.7%)	135 (44.6%)		259 (48.5%)
Sex			0.737			0.001	
Male	183 (75.6%)	216 (74.0%)		156 (67.5%)	243 (80.2%)		399 (74.7%)
Female	59 (24.5%)	76 (26.0%)		75 (32.5%)	60 (19.8%)		135 (25.3%)
Size (cm) ^2^	4.0 [3.0–6.0]	5.5 [3.8–8.8]	<0.001 ^3^	4.0 [3.0–6.5]	5.3 [3.9–8.0]	<0.001 ^3^	4.8 [3.2–7.5]
TNM stage			<0.001			<0.001	
Low (I or II)	204 (84.3%)	180 (61.6%)		191 (82.7%)	193 (63.7%)		384 (71.9%)
High (III or IV)	38 (15.7%)	112 (38.4%)		40 (17.3%)	110 (36.3%)		150 (28.1%)
WHO grade			0.085			<0.001	
Low (1 or 2)	140 (57.9%)	146 (50.0%)		155 (67.1%)	131 (43.2%)		286 (53.6%)
High (3 or 4)	102 (42.1%)	146 (50.0%)		76 (32.9%)	172 (56.8%)		248 (46.4%)

^1^ pAMPK-positive tumor extent >10% ^2^ Median with 25–75% quartile ^3^ Mann–Whitney U test. The other p-values were calculated by Pearson’s χ^2^ test. Abbreviation: Cyt, cytoplasm; Pos, positive; Neg, negative; Nuc, nucleus.

**Table 2:** Cox regression analyses for pAMPK expression of the discovery cohort.

Analysis Detail	Progression-Free Survival		Overall Survival		Cancer-Specific Survival	
	HR (95% CI)	*p*	HR (95% CI)	*p*	HR (95% CI)	*p*
Univariate analysis						
pAMPK-C (Pos vs Neg)	0.190 (0.110–0.310)	<0.001	0.470 (0.320–0.680)	<0.001	0.200 (0.110–0.370)	<0.001
pAMPK-N (Pos vs Neg)	0.140 (0.080–0.260)	<0.001	0.440 (0.300–0.650)	<0.001	0.070 (0.030–0.19)	<0.001
TNM stage (≥III vs ≤II)	12.920 (8.150–20.490)	<0.001	5.480 (3.790–7.920)	<0.001	18.050 (10.100–32.270)	<0.001
WHO Grade (≥3 vs ≤2)	5.210 (2.980–9.120)	<0.001	2.770 (1.850–4.160)	<0.001	16.330 (5.930–44.950)	<0.001
Multivariate analysis						
pAMPK-C (Pos vs Neg)	0.260 (0.153–0.442)	<0.001	0.656 (0.446–0.965)	0.032	0.374 (0.205–0.681)	0.001
TNM stage (≥III vs ≤II)	8.644 (5.340–13.992)	<0.001	4.163 (2.806–6.178)	<0.001	9.535 (5.245–17.336)	<0.001
WHO Grade (≥3 vs ≤2)	2.601 (1.456–4.646)	0.001	1.774 (1.156–2.724)	0.009	7.163 (2.552–20.106)	<0.001
Multivariate analysis						
pAMPK-N (Pos vs Neg)	0.308 (0.159–0.595)	<0.001	0.767 (0.500–1.177)	0.225	0.232 (0.090–0.600)	0.003
TNM stage (≥III vs ≤II)	7.944 (4.868–12.965)	<0.001	4.250 (2.850–6.337)	<0.001	8.677 (4.754–15.837)	<0.001
WHO Grade (≥3 vs ≤2)	1.889 (1.024–3.487)	0.042	1.696 (1.082–2.660)	0.021	5.086 (1.777–14.556)	0.002

Abbreviation: HR, hazard ratio; CI, confidence interval; C, cytoplasm; Pos, positive; Neg, negative; N, nucleus.

**Table 3:** Cox regression analyses for pAMPK expression of the validation cohort.

Analysis Detail	Progression-Free Survival		Overall Survival		Cancer-Specific Survival	
	HR (95% CI)	*p*	HR (95% CI)	*p*	HR (95% CI)	*p*
Univariate analysis						
pAMPK–C (Pos vs Neg)	0.250 (0.180–0.360)	<0.001	0.480 (0.340–0.690)	<0.001	0.180 (0.100–0.310)	<0.001
pAMPK–N (Pos vs Neg)	0.300 (0.210–0.440)	<0.001	0.350 (0.230–0.510)	<0.001	0.180 (0.100–0.330)	<0.001
TNM stage (≥III vs ≤II)	6.430 (4.720–8.760)	<0.001	4.740 (3.390–6.620)	<0.001	10.340 (6.570–16.270)	<0.001
WHO Grade (≥3 vs ≤2)	3.010 (2.190–4.140)	<0.001	2.870 (2.020–4.080)	<0.001	4.640 (2.880–7.470)	<0.001
Multivariate analysis						
pAMPK–C (Pos vs Neg)	0.304 (0.210–0.441)	<0.001	0.629 (0.438–0.903)	0.012	0.256 (0.144–0.455)	<0.001
TNM stage (≥III vs ≤II)	4.630 (3.352–6.395)	<0.001	3.567 (2.505–5.079)	<0.001	6.446 (4.023–10.328)	<0.001
WHO Grade (≥3 vs ≤2)	2.244 (1.618–3.112)	<0.001	2.091 (1.453–3.010)	<0.001	2.935 (1.801–4.781)	<0.001
Multivariate analysis						
pAMPK–N (Pos vs Neg)	0.405 (0.280–0.585)	<0.001	0.471 (0.315–0.705)	<0.001	0.296 (0.164–0.536)	<0.001
TNM stage (≥III vs ≤II)	4.989 (3.617–6.883)	<0.001	3.601 (2.537–5.111)	<0.001	7.101 (4.434–11.371)	<0.001
WHO Grade (≥3 vs ≤2)	1.882 (1.350–2.623)	<0.001	1.844 (1.274–2.667)	0.001	2.344 (1.430–3.844)	<0.001

Abbreviation: HR, hazard ratio; CI, confidence interval; C, cytoplasm; Pos, positive; Neg, negative; N, nucleus.

## Supplementary Materials

The following are available online at https://www.mdpi.com/2072-6694/11/10/1602/s1, Figure S1: Establishment of the cut-off criteria for pAMPK positivity.
