# Supplementary material for: Prognostic Implication of pAMPK Immunohistochemical Staining by Subcellular Location and Its Association with SMAD Protein Expression in Clear Cell Renal Cell Carcinoma
Source: Cancers (Basel). 2019 Oct 21;11(10):1602. doi: 10.3390/cancers11101602 (PMC6826619; doi:10.3390/cancers11101602)
Supplement: Supplementary file 1 [file cancers-11-01602-s001.pdf]

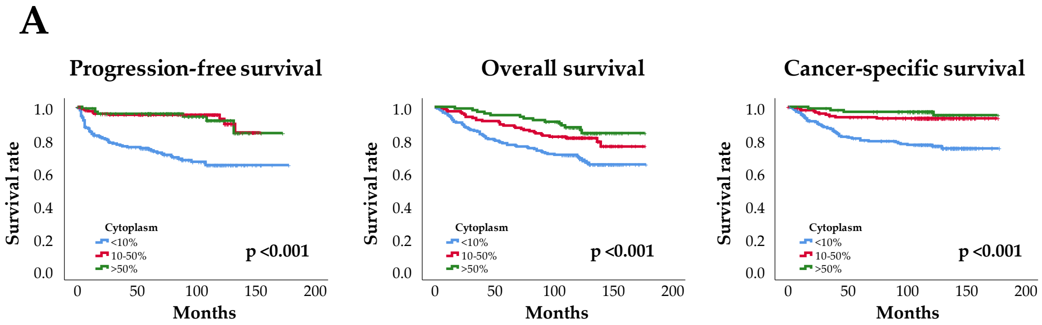

Cytoplasmic staining extent	<10%	10-50%	>50%
Case number	203	150	100

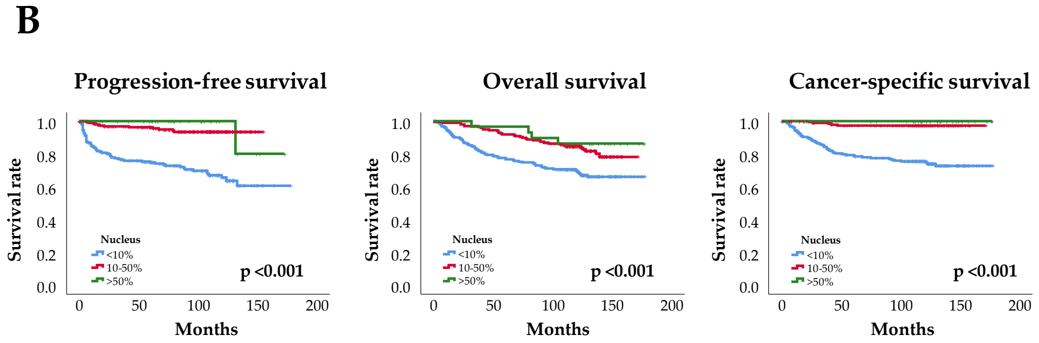

Nuclear staining extent	<10%	10-50%	>50%
Case number	225	198	30

Figure S1. Establishment of the cut-off criteria for pAMPK positivity. First, prognosis of ccRCC was evaluated according to the pAMPK-staining extent (<10%; 10-50%; >50%) for both **(a)** cytoplasmic and **(b)** nuclear staining in the discovery cohort. In both localization of pAMPK expression, tumors showing <10% expression showed distinctly poor outcomes. Based on the distribution of the cases and the prognostic implications, therefore, pAMPK-positivity in ≥10% of tumor cells was set as the cutoff.
